# Current trends and prospects of surgical techniques for hepatoblastoma

**DOI:** 10.1002/cam4.6795

**Published:** 2024-01-05

**Authors:** Jia‐rui Pu, Hang Li, Shuai Li, Yong Wang, Shi‐wang Li, Shao‐tao Tang

**Affiliations:** ^1^ Department of Pediatric Surgery, Union Hospital, Tongji Medical College Huazhong University of Science and Technology Wuhan China; ^2^ Department of Gastrointestinal Surgery, Union Hospital, Tongji Medical College Huazhong University of Science and Technology Wuhan China

**Keywords:** advances, hepatoblastoma, prospects, surgical technology

## Abstract

Hepatoblastoma, a common extracranial malignant solid tumor in childhood, is often detected at an advanced stage and is difficult to treat surgically. Despite the availability of multiple comprehensive treatments that can be combined with surgery, hepatoblastoma treatment outcomes remain poor. Surgery is the main treatment strategy for hepatoblastoma, but it faces many challenges, including tumor attachment to surrounding tissues, tumor wrapping or invading of vital organs and tissues, the presence of giant or multiple tumors, distant metastasis, the formation of a tumor thrombus, and significant surgical trauma. In this review, we discuss recent research advances and propose potential strategies for overcoming these challenges. Such strategies may improve the rate of hepatoblastoma resection and local control in children, as well as reduce complications and trauma.

## 1 INTRODUCTION

Hepatoblastoma is a common extracranial malignant solid tumor in childhood that is often discovered at an advanced stage. It is associated with a physical condition and is difficult to treat, resulting in poor outcomes, and the 5‐year overall survival rate of advanced hepatoblastoma is lower than 50%.^1^

In recent years, there have been significant advances in surgical techniques and other comprehensive treatment methods for childhood hepatoblastoma, which have improved their efficacies. Preoperative chemotherapy and radiotherapy can reduce tumor size, clinical stages, intraoperative bleeding, and local recurrence rate, making surgical resection easier. Presently, preoperative neoadjuvant chemotherapy and radiotherapy, where necessary, are advocated for advanced hepatoblastoma. Even so, surgical treatment continues to face significant challenges, including the following: (1) Inability to accurately plan surgery because of the complexity of advanced disease. (2) Adhesions between the tumor and its surrounding tissues, which makes it hard to identify and separate the tumor. (3) The presence of very large tumors, multiple tumors, or tumors in vital organs, which are difficult to completely remove. (4) Tumors that surround or invade large blood vessels may cause fatal, massive hemorrhage upon removal. (5) Intravascular or even cardiac tumor thrombosis may have serious consequences, including pulmonary embolism. (6) Surgery, or the tumor itself, may irreversibly damage or functionally impair vital organs. (7) The operation may cause significant trauma and scarring, which may cause disfigurement. Here, we summarize recent advances in the surgical treatment of hepatoblastoma, aiming to improve the therapeutic effect and prognosis.

## 2 SURGERY PLANNING AND SURGICAL NAVIGATION

### 2.1 Virtual surgery platform

The virtual surgery platform relies on medical image data to computationally process and reconstruct tissue models. It can simulate the resection of complex tumors, calculate the volume of remaining organs, and evaluate the tumor resectability. In children, the virtual surgery system has been used for various abdominal malignant tumors and is most widely used to treat hepatoblastoma. It can segment the liver before surgery, accurately determine tumor size and invasion range, perform simulated surgical cutting, evaluate postoperative liver size and function, and assess surgical resectability.

The surgical planning system used by Warmann et al.^2^ enabled 85% of patients with complex hepatoblastoma to undergo extended hepatectomy, avoiding liver transplantation and achieving a therapeutic effect that was equivalent to that of liver transplantation. One of the most important indices of preoperatively evaluating the tumor is residual organ volume, which is achieved using a virtual surgery system to determine the resection line, determine residual organ volume, and simulate radical tumor resection before surgery. This cannot be done through preoperative imaging techniques, such as magnetic resonance imaging (MRI).^3^ Because not all lesions can be displayed during preoperative imaging assessment, virtual resection plans differ from actual operations, and further research is needed to minimize the differences. In addition, calculating the residual organ volume alone is not sufficient for comprehensive preoperative evaluation, and the clinical relevance of the virtual resection platform needs to be strengthened by combining predictive indices such as postoperative organ function and surgical complications.

### 2.2 3D printing technology

Three‐dimensional (3D) printing software uses preoperative imaging examination data for 3D visualization and then prints tumors and adjacent tissues and organs using different materials. Compared with imaging examination results, such as MRI data, 3D printing enables surgeons to more intuitively understand the relationship between tumors and surrounding tissues and to perform preoperative simulation operations.^4^, ^5^ Sánchez et al. used real‐time 3D printing before and during surgery to perform a more thorough and safer resection of complex tumors. About 15% of complicated hepatoblastoma cases cannot be surgically removed after chemotherapy and need liver transplantation.^6^ Using 3D printing on patients with PRETEXT IV hepatoblastoma, whose tumors were located in the porta hepatis, Souzaki et al.^7^ revealed the relationship between the tumor and the portal vein and hepatic vein, which enabled smooth surgery without complications and avoided liver transplantation. Yang et al.^8^ have proposed the use of a 3D model to help parents understand liver tumor anatomy, surgical planning, and surgical risks, as well as to improve the doctor–patient relationship. However, because 3D printing involves at least 4–5 h, it is not feasible for real‐time surgical applications. Moreover, it cannot easily model some scenarios, such as bleeding and adhesion. In addition, further research is needed to improve printing accuracy, optimize printing materials, and standardize image acquisition for 3D model printing.

### 2.3 Intelligent surgical navigation

With the development of digital intelligent technology, intelligent surgical navigation technologies such as augmented reality, mixed reality, and artificial intelligence have emerged, which might improve surgical positioning accuracy, reduce surgical injuries, optimize surgical paths, and improve surgical success rates. Intelligent navigation is also used in the treatment of intractable abdominal tumors in children, such as hepatoblastoma and neuroblastoma. Dong et al. reported the use of an intelligent navigation system based on CT scanning to resect hepatoblastoma, including complex cases. By preoperatively acquiring CT digital signals and using iterative reconstruction and other procedures, it can intraoperatively control the size and direction of a virtual 3D liver model in real‐time using gestures intraoperatively, and improve the accuracy of anatomical hepatectomy, thereby reducing surgical injury and postoperative hospitalization time.^9^ The surgeon can zoom in and out on the 3D image at any time during the surgery, which is not possible with preoperative imaging examinations such as MRI.

However, intelligent navigation faces several challenges, such as tissue deformation caused by abdominal breathing of children, pneumoperitoneum, or tissue traction, which affect the similarity between preoperative images and the actual situation during surgery. Teatini et al.^10^ suggested using intraoperative CT imaging and user‐independent registration methods to compensate for tissue deformation. Pelanis et al. proposed a method of obtaining CT images in the mixed operating room and optimizing the navigation update scheme. Another disadvantage of virtual 3D model is the lack of tactile feedback, which can be used in some cases.^11^ Therefore, further research should develop real‐time navigation methods that improve the navigation system's accuracy and compensate for tissue and organ deformation.

### 2.4 Tumor tracer

Complete resection is crucial for the treatment of pediatric tumors. However, judging the edge of the lesion with the naked eye is often difficult, and distant metastases are hard to detect, which prevents tumor resection. Moreover, preoperative MRI and other magnetic resonance imaging assessments do not provide real‐time lesion visualization. Indocyanine green (ICG), a tumor‐tracing fluorescence agent, specifically aggregates in some tumor tissues, and when stimulated with near‐infrared light, an optical image of tumor and normal tissues can be visualized. In recent years, ICG has been applied to detect childhood tumors. Abdelhafeez et al.^12^ determined the safe dose of ICG in pediatric tumor patients and showed that ICG‐guided fluorescence navigation is feasible and safe for most pediatric solid tumors, including neuroblastoma and hepatoblastoma. At present, ICG is widely used in hepatoblastoma, especially to visualize lung metastases, which are difficult to identify with the naked eye.^13^, ^14^ Lake et al.^15^ reported that the identification of primary and metastatic hepatoblastoma using ICG fluorescence navigation has a sensitivity of 91% and that it can reveal metastatic tumors that were not detected using preoperative imaging, thereby reducing the need to use thoracotomy to locate tumors. However, they suggested that the use of ICG may be limited in cases of multiple and deep lesions. Yamamichi et al. reported that preoperative CT‐guided injection of indigo carmine dye into the tumor focus was used for tumor localization and that its intraoperative combination with ICG navigation can identify small metastatic lesions. The smallest diameter of the identified tumor foci was 1 mm, and their average diameter was 3 mm.^16^

These reports indicate that using ICG fluorescence navigation to trace tumors in children can effectively detect the tumor focus and metastasis, thereby improving the rate of complete tumor resection and reducing recurrence. The application limitations of ICG in hepatoblastoma include low specificity, high background signal, difficulty in detecting deep lesions, and a high false‐positive rate. New tracing materials for pediatric tumors can be developed to improve the specificity of the tracer, detect the lesions more accurately and completely, and facilitate R0 resection.

## 3 MULTIPLE OR MULTISITE TUMORS

Because malignant tumors are often not confined to one organ or body cavity, complete lesions resection may be traumatic or difficult to achieve. Although organ transplantation can aid in complete tumor resection, it has disadvantages, such as long wait times for suitable donors, a need for lifelong use of antirejection drugs, and a high economic cost. Pediatric surgeons can completely remove these complex tumors and minimize surgical trauma through various strategies, like improving the surgical approach and resection sequence.

Associated liver partition and portal vein ligation for staged hepatectomy (ALPPS), which have been successfully used on adult patients, can avoid severe hepatic insufficiency caused by low postoperative residual liver volume. Its first stage involves the separation and ligation of the right portal vein and separate of the liver in situ, so that the affected liver atrophies while portal vein blood flow to the normal liver and normal liver proliferation occur rapidly. In the second stage, after the normal liver has grown to more than 40% in one to 2 weeks, the diseased liver is resected.^17^

In recent years, this technique has also been used to treat pediatric liver tumors, such as hepatoblastoma with large tumor bodies or multiple parts. Wiederkehr et al.^18^ reported that five liver cases that were considered unresectable using traditional methods were safely and effectively removed using ALPPS without total hepatectomy or liver transplantation. Hong et al. successfully operated on a 54‐day‐old infant with hepatoblastoma using a modified ALPPS procedure. Because the tumor affected liver segments IV, V, VI, VII, and VIII, the estimated residual liver volume was insufficient.^19^ On day 7, after the first stage of the surgery, the residual liver volume had increased by 91%. The second stage of the surgery achieved R0 resection, and the child recovered well after the operation, indicating that the modified ALPPS procedure was feasible and safe in infants. Akadzed et al.^20^ described the treatment of a 20‐month‐old infant with hepatoblastoma using ALPPS, in which the first stage was completed using laparoscopy, highlighting that laparoscopy is feasible for this complex operation. The liver volume of children is reported to increase by 46%–91% between day 8 and 16 after the first stage of the operation.^17^ Although the liver regeneration potential is strong in children, there are no guidelines on the length of interval between the first and second stage of ALPPS. Whether ALPPS protocols for adults and children follow the same principle warrants further exploration.

**FIGURE 1:**
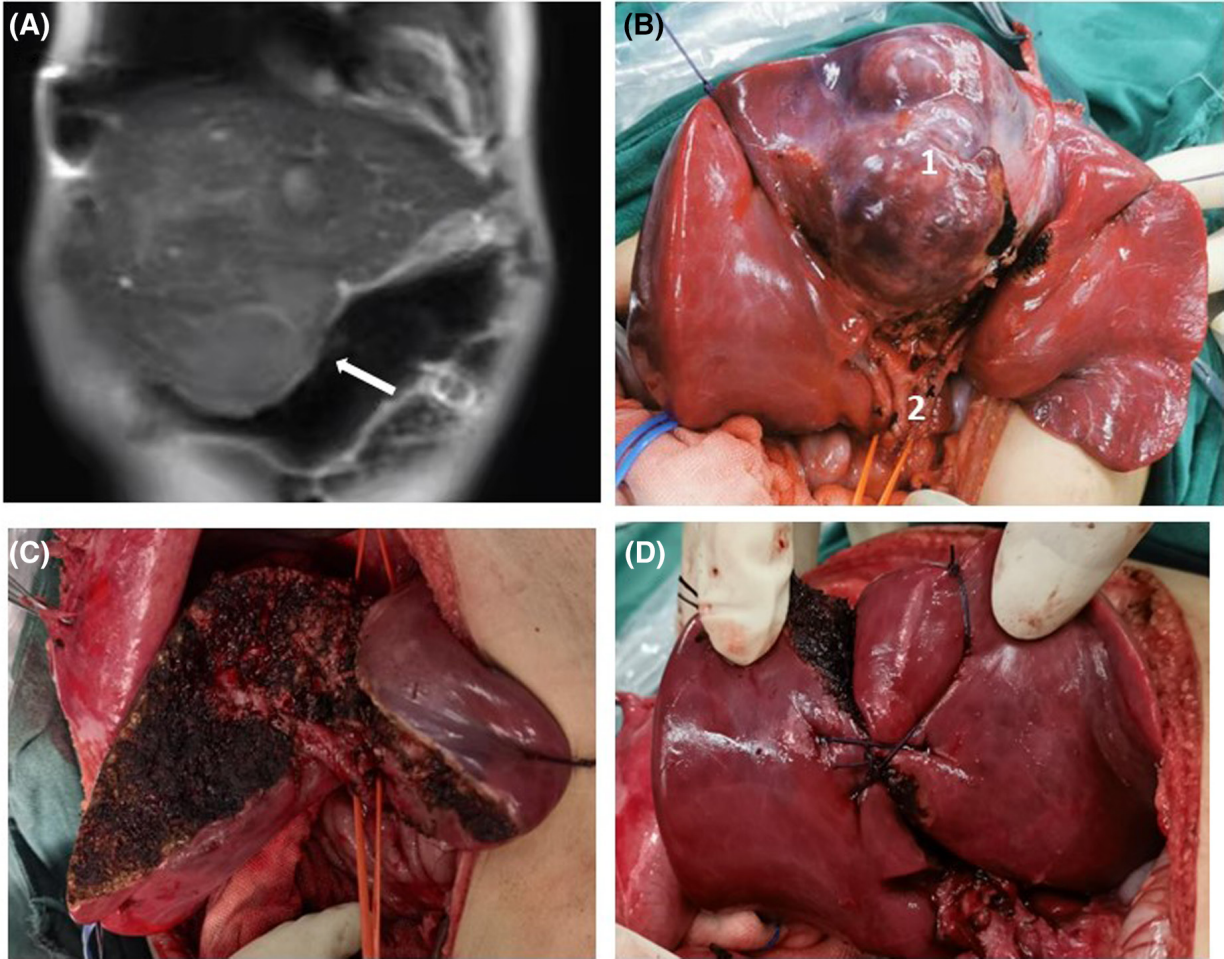
Middle‐lobe hepatectomy on a 9‐month‐old patient with central hepatoblastoma: (A) Magnetic resonance imaging revealed a central liver tumor in an infant. (B) 1 giant liver tumor, 2 anatomy of the first hepatic hilum. (C) Complete resection of the liver tumor. (D) Reconstruction of remaining liver tissue.

Central hepatectomy (CH) is used to resect liver tumors located in the center and can avoid extensive hepatectomy or liver transplantation while preserving as much normal liver parenchyma as possible to minimize the risk of postoperative liver failure. However, it requires advanced surgical techniques, especially in children, and a highly accurate anatomy of key liver parts, such as the liver hilum. We successfully performed a middle‐lobe hepatectomy on a 9‐month‐old patient with central hepatoblastoma and completely removed the tumor without biliary fistula or other complications (Figure 1).

## 4 TREATMENT OF TUMORS SURROUNDING OR INVADING LARGE BLOOD VESSELS

Complex hepatoblastoma often closely associate with and displace large blood vessels. These tumors may even wrap around or invade large blood vessels, which can impede tumor resection or cause serious complications. The major vessels involved are the abdominal aorta, inferior vena cava, and renal vessels, among which the inferior vena cava is the most frequently involved. In the past, the wrapping or invasion of large blood vessels was considered a taboo in radical surgery. However, with advances in surgical techniques, especially vascular surgery, it is possible to completely resect such tumors.^21^

### 4.1 Vascular repair

Vascular repair can only be carried out in cases involving the partial resection of a vascular wall or partial injury of large vessels. Direct end‐to‐end anastomosis after vascular resection is suitable for cases in which residual vessels are sufficiently long. However, because the length is often insufficient for direct anastomosis, vascular replacement is needed. In situ repair is mainly used to correct small vascular wall defects. Some studies indicate that the loss of the internal diameter of the inferior vena cava by less than 20% will not affect the patency of blood vessels.^22^ If in situ repair leads to lumen stenosis and affects blood flow, patch repair may be required. Patch source materials include autologous blood vessels, the pericardium, the peritoneum, or artificial materials.^23^, ^24^ Although small vascular defects can be repaired using the above‐mentioned methods, vascular replacement should be considered for extensive vascular defects or when long blood vessels need to be removed.

### 4.2 Vascular replacement

Extensive vascular involvement requires the use of synthetic, autologous grafts or xenografts to reconstruct blood vessels. Sarti published the first report on the replacement of the inferior vena cava with polytetraflfluoroEthylene (PTFE) graft in 1970, and this was followed by reports of using various vein or artery grafts. Currently, the great saphenous vein, PTFE, and pericardium can be used for this purpose. Some scholars have reported the use of bovine pericardium to reconstruct the inferior vena cava.^23^ Because children are undergoing growth and development, the use of artificial blood vessels as replacements is challenging.

Angelico et al. reported a hepatoblastoma case that was considered unresectable using traditional techniques, and the inferior vena cava remained tumor‐infiltrated after chemotherapy. Thus, the authors performed an orthotopic hepatectomy under hypothermic cardiopulmonary bypass and replaced the inferior vena cava using a donated aorta from a deceased individual with the same blood type. After an 8‐month follow‐up, the liver function of the child was normal, and there were no complications in vascular transplantation.^25^ Trends in future research include developing new vascular materials, which can improve the histocompatibility of materials and adapt to children's growth and development.

## 5 MANAGEMENT OF TUMOR THROMBUS IN BLOOD VESSEL AND/OR HEART

Hepatoblastoma is often accompanied by tumor thrombi in important blood vessels, which most often involves the inferior vena cava. Tumor thrombi can extend to the inferior vena cava and right atrium and may even lead to heart failure and dyspnea, which makes treatment complex and challenging. Preoperative neoadjuvant chemotherapy can reduce or clear some tumor thrombi, as well as reduce the risk of surgery. However, surgical intervention may be necessary for tumor thrombi that are unchanged after multiple intensive treatments. Methods of accurate preoperative tumor thrombus evaluation, such as tumor thrombus classification, the establishment of collateral circulation, the risk of embolism, and standardized surgical procedures (such as the order of thrombus removal, whether to perform cardiopulmonary bypass, and the possibility of minimally invasive surgery), warrant further investigation.

### 5.1 The relationship between tumor thrombus and diaphragm plane

Tumor emboli, which are generally divided into infrahepatic, retrohepatic, suprahepatic, and atrial tumor thrombi, can also be classified based on their relationship with the diaphragm plane. Fanelli et al. proposed a strategy for treating childhood abdominal tumors with tumor thrombi complications. Using their strategy, extracorporeal circulation does not need to be established for subphrenic tumor thrombi, which can be removed by first controlling and transecting the blood vessels. Alternatively, part of the involved blood vessels can be removed for vascular reconstruction or replacement.^26^ For tumor thrombi at the diaphragm level, extracorporeal circulation should be established, whereas tumor thrombi above the diaphragm require surgery under cardiopulmonary bypass with cardiac arrest. Depending on the time of cardiac arrest, this procedure should be carried out at low temperature. In cases involving long operations, selective cerebral perfusion is needed. However, because hypothermia is harmful to children, its frequency and duration should be minimal.

### 5.2 The need for establishing collateral circulation

To avoid serious complications like pulmonary embolism, which can be caused by tumor thrombus detachment, the disconnected inferior vena cava can be completely removed. However, insufficient preoperative evaluation and temporary inferior vena cava may be associated with risks, such as an insufficient blood supply. Therefore, the need for establishing collateral circulation should be fully evaluated before surgery. With good collateral circulation, the inferior vena cava can be closed below the hepatic vein to allow the removal of the tumor and inferior hepatic vena cava as a whole.^27^

## 6 LIVER TRANSPLANTATION

Although surgical resection is the main treatment method for treating hepatoblastoma, comprehensive treatment can improve the outcomes of malignant tumors. Because the best outcomes of surgical treatment of malignant tumors are achieved with R0 resection, traditional surgical treatment may cause irreversible injury or ischemia in vital organs. Organ transplantation offers an effective strategy for overcoming this challenge.

### 6.1 Liver transplantation

For hepatoblastoma treatment, the first choice is to remove the tumor and the involved liver. However, 60% of patients present with unresectable tumors, and even after chemotherapy, 15% of the cases remain unresectable. In such cases, liver transplantation is needed. The main indications for liver transplantation are PRETEXT stage IV and POST‐TEXT stage III with hepatic vein or portal vein involvement. Studies indicate that for such patients, total hepatectomy combined with orthotopic liver transplantation can achieve a five‐year overall survival rate of 80%–90%. Moreover, living donor liver transplantation for such patients has been proven to be technically safe for donors. Advances in technology have expanded the indications for liver transplantation. Some scholars have performed liver transplantation on hepatoblastoma patients following the resection of multiple lung metastases. Lung metastases reappeared 5 months after liver transplantation, and the 2‐year survival rate reached 91%.

Shimizu et al. reported using the whole liver and tumor thrombus removal under a cardiopulmonary bypass, followed by living donor liver transplantation in hepatoblastoma cases in which tumor thrombi extended to the inferior vena cava and the right atrium.^28^, ^29^, ^30^, ^31^ Due to the shortage of donor livers and the need for the long‐term use of antirejection drugs, allogeneic liver transplantation has been applied to complex pediatric liver tumors that are not suitable for routine liver resection. This method does not require waiting for the liver source or using antirejection drugs for a long time, and the economic cost is lower.^32^ The most common reason for the death of patients with hepatoblastoma after transplantation is tumor progression, especially for patients who underwent hepatectomy for the first time and liver transplantation for the second time. Tumor progression is more common, and the use of chemotherapy after transplantation is controversial. Some studies have shown that the mortality rate of salvage liver transplantation is higher than that of initial liver transplantation, while others show that the mortality rates of the two groups are similar. This suggests that patients who recur after tumor resection can undergo a second resection surgery using methods like ex‐vivo resection and autologous transplantation without the need for urgent liver transplantation.^33^ Therefore, further investigations are needed to establish the indications and timing of liver transplantation in hepatoblastoma patients, as well as to determine if chemotherapy should be given after surgery.

### 6.2 Multivisceral transplantation

In 2015, Samuk reported the case of a child with recurrent hepatoblastoma who underwent multiple organ transplantation (MVTx). The patient underwent a right hemihepatectomy at the age of 1 year and suffered from intestinal ischemia, which caused a short bowel. However, the tumor recurred after 16 months with portal vein involvement. The patients then underwent liver, pancreas, stomach, small intestine, and large intestine transplantation without experiencing major early post‐operative complications.^34^ Multiorgan transplantation can achieve the complete gross resection of intra‐abdominal malignant tumors in patients with multiorgan and vascular involvement. However, it is hard to find donors for multiorgan transplantation. Moreover, the immune rejection of allogeneic multiorgan transplantation is more likely to occur, and the success rate of transplantation may be lower. Thus, this type of surgery requires the repeated assessment of advantages and disadvantages and is usually reserved for cases with no other feasible alternative treatments. At present, few studies have described multiple organ transplantation for the treatment of hepatoblastoma, and further research is needed in this area.

## 7 MINIMALLY INVASIVE/ROBOTIC‐ASSISTED SURGERY

Minimally invasive surgery, which is widely used to treat adult tumors and benign childhood tumors, is characterized by short hospital stays, minimal trauma, and good cosmetic effect. However, open surgery is still the main treatment for childhood hepatoblastoma. Because the surgical space is smaller in children and children poorly tolerate long‐term surgical anesthesia, tumor dissemination during surgery should be prevented. Currently, the application of minimally invasive surgery to treat hepatoblastoma is controversial. Kwon et al.^35^ reported that the safe use of laparoscopic liver resection in 13 hepatoblastoma cases and suggested that the main strategies for minimizing complications include a thorough preoperative evaluation of the resection plane, major blood vessels, and hilar anatomy.

Robotic surgery allows 3D visualization and flexible surgery, and it has been used to treat advanced abdominal malignant tumors in adults. For example, Professor Zhang Xu's robotic surgery for renal malignant tumors with tumor thrombi highlighted various surgical strategies for removing tumor thrombi of different grades. Thus, robotic surgery can be successfully used for very complex and dangerous tumors.^27^, ^36^ However, some studies indicate that malignant tumors that cross the midline are contraindications for robotic surgery. Additionally, performing extensive lymph node dissection using robotic surgery is difficult and may take long time to complete in case of massive hemorrhage. The use of robotic surgery in young and underweight children is also limited. At present, robotic surgery has limited applications and is rarely used for advanced tumors. Blanc et al.^37^, ^38^ conducted a nationwide prospective study on robotic surgery for pediatric malignant tumors, which showed that robotic surgery is shorter than open surgery for some malignant tumors, under the premise of ensuring safety. Chen et al.^39^ reported the successful use of robot‐assisted gallbladder‐preserving hepatectomy to treat childhood S5 hepatoblastoma. We also believe that robotic surgery is beneficial to perform delicate operations such as suturing in deeper parts and smaller spaces, as well as carry out tissue reconstruction after tumor resection in children.^40^

At present, the safety and effectiveness of new energy devices have been proven by multiple studies. These multifunctional anatomical devices can achieve rapid dissection and accurate hemostasis, as well as perform complex laparoscopic or robotic surgeries, thereby reducing the surgical conversion rate caused by bleeding and expanding the indications of minimally invasive or robotic hepatoblastoma surgery. Based on the physiological characteristics of children, surgical instruments that are suitable for limited spaces, as well as surgical procedures and techniques that suit children. Minimally invasive and robotic surgery would provide more treatment methods for childhood hepatoblastoma.

## 8 CONCLUSION

**FIGURE 2:**
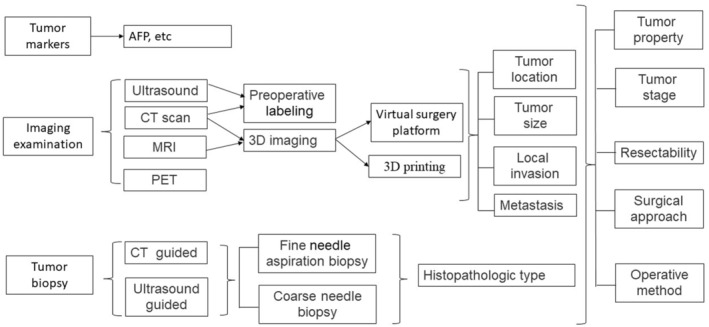
Flow chart for the diagnostic approach and surgical planning for hepatoblastoma.

**FIGURE 3:**
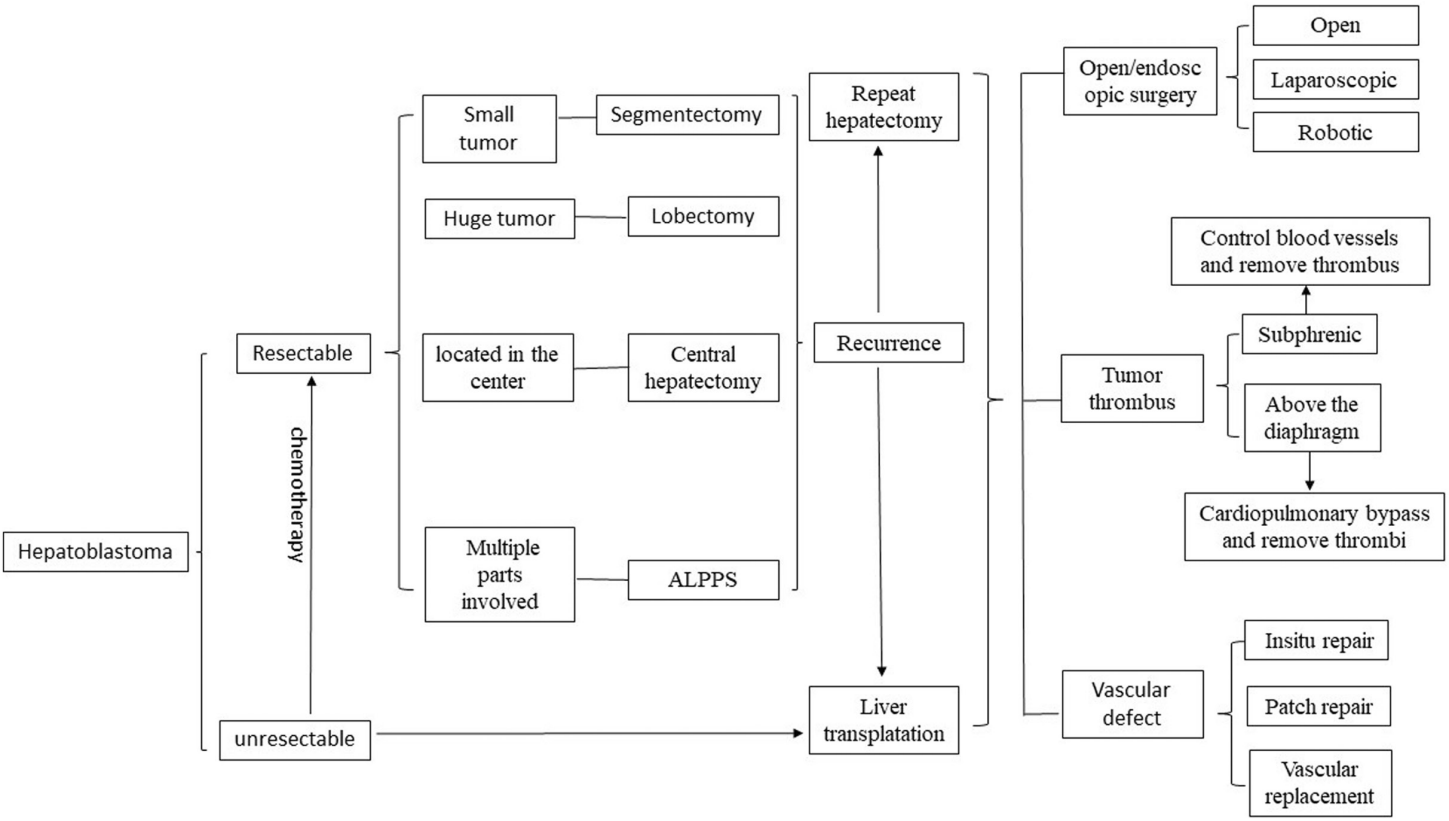
Mind map to illustrate surgical techniques for hepatoblastoma.

The diagnoses and surgical techniques for hepatoblastoma have developed rapidly (Figure 2 and Figure 3 are respectively used to illustrate the relevant diagnoses and surgical techniques mentioned above), and the overall therapeutic effect has been significantly improved through multidisciplinary, comprehensive treatment. However, when compared with adult surgery, the technical development of pediatric tumor surgery still lags. At present, medicine and engineering disciplines are increasingly cross‐integrated. As physicians continue to improve their surgical skills, the simultaneous emergence of new biomaterials, artificial intelligence, big data, and related technology will markedly improve the accuracy and effectiveness of hepatoblastoma surgeries. These advancements may also improve the accuracy of diagnosing and treating childhood hepatoblastoma at the cellular and molecular level, which may improve prognosis and the quality of life. This review can guide surgical planning and surgical technique selection for hepatoblastoma treatment, especially in complicated cases.

## AUTHOR CONTRIBUTIONS

**Jia‐rui Pu:** Data curation (lead); formal analysis (lead); writing – original draft (lead); writing – review and editing (lead). **Hang Li:** Data curation (lead); software (lead). **Shuai Li:** Investigation (supporting); methodology (supporting); visualization (supporting). **Yong Wang:** Data curation (supporting); investigation (supporting); methodology (supporting). **Shi‐wang Li:** Writing – original draft (equal); writing – review and editing (equal). **Shao‐tao Tang:** Formal analysis (lead); supervision (lead); writing – review and editing (lead).

## FUNDING INFORMATION

This work was supported by the National Natural Science Foundation of China (Grant/Award Number 81772967, 82372924), Natural Science Foundation of Hubei Province (Grant/Award Number 2019CFB496).

## CONFLICT OF INTEREST STATEMENT

The authors have no conflicts of interest to disclose.

## ETHICS STATEMENT

The Ethics Committee of Union Hospital, Tongji Medical College, Huazhong University of Science and Technology approved this study. The approval number is [2016]lsz(s180). As a result of retrospective study, the requirement of informed consent was exempted by the ethics committee.

## Data Availability

All relevant data are within the manuscript and its additional files.
